# Quantitative correlations between soil and plants in reclaimed mining dumps using a coupling coordination degree model

**DOI:** 10.1098/rsos.180484

**Published:** 2018-09-19

**Authors:** Anning Guo, Zhongqiu Zhao, Ye Yuan, Yangyang Wang, Xuezhen Li, Ruicong Xu

**Affiliations:** 1 College of Land Science and Technology, China University of Geosciences, No. 29, Xueyuan Road, Haidian District, Beijing 100083, People's Republic of China; 2 Key Laboratory of Land Consolidation and Rehabilitation Ministry of Land and Resources, Beijing 100035, People's Republic of China

**Keywords:** opencast mine, soil, plant, reclamation patterns, coupling coordination degree

## Abstract

It is generally accepted that coevolution between soil and plant has great significance for the sustainable development of mining dumps in fragile eco-environment. However, this was not very clear in opencast mine area located in Western China. Based on comprehensive index systems and a combination of subjective and objective weighting method, a coupling coordination degree model, including comprehensive evaluation function, coupling degree and coupling coordination degree, was established to find the ‘short plank’ of different reclamation patterns and to quantify the status quo of coevolution between soil and plant systems in mined plots. The results indicated that only the plot with *Pinus tabuliformis* was under synchronous development, a mixed model of *Robinia pseudoacacia*–*Pi. tabuliformis* and *R. pseudoacacia* monoculture were developed with vegetation lagging, while plots *R. pseudoacacia*–*Ulmus pumila*–*Ailanthus altissima* and original landform were soil lagged. All plots were in the state of primary and intermediate coordination. Thus, some effective measures should be taken for the further development in different patterns.

## 1. Introduction

Coal, widely regarded as black gold and food for industry, has been a major resource which makes huge contributions to the daily production of China [1]. To meet the energy consumption, mining industry has been largely promoted in the last two decades of the twentieth century [2]. This would surely be beneficial to the whole economy. However, the environment has been destroyed a lot at the same time because of this human activity. Opencast mining needs to firstly eliminate vegetation, remove soil and overburden by excavation, and this would inevitably change the topography and geological structures, disrupt surface and subsurface hydrologic regimes and further reshape the landforms [3], and soils are also highly compacted by the frequent movement of heavy equipment [4]. To our knowledge, 8.84 × 10^3^ hm^2^ of disturbed land and 1.63 × 10^4^ hm^2^ of waste dumps have been produced in China and are still increasing at a rate of 8–9% annually [5]. Researchers estimated that it might take approximately 200 years of natural succession for total nitrogen pool to recover to the original level [6], all of which make it a great challenge to maintain the balance of ecosystem, especially in such a fragile region in Western China.

Considering that it is difficult for natural succession in these districts owing to the restriction of soil degradation, some effective measures are of essential importance to recover the ecosystem [7]. Revegetation, a common method adopted by many countries such as USA, China and India, has been proved to be a proper reclamation way to accelerate the soil-forming processes, build up soil organic matter, develop nutrient cycling and restore soil productivity [8,9]. According to Fu [10], physico-chemical properties of mine soil in Inner Mongolia of China have improved a lot after 15-year reclamation. However, not all trees are of the same efficiency. The success of reclamation also hinges on the type of plant selected [11–13]. Dutta & Agrawal [14] did research about five exotic tree species planted on coal mine spoils in India and found significant difference in the improvement of soil properties.

The coevolution of soil and pant is the most pressing question in reclaimed areas. Scholars have conducted a large number of in-depth researches into the evolution of vegetation or soils. Juwarkar *et al.* [12] observed physico-chemical status of mine land which had been reclaimed for about 20 years in India and found that soil organic carbon pool increased from 0.104 to 0.69% in 0–15 cm spoil depth. Keskin & Makineci did a research in two different reclaimed spoils in Turkey, of which one was black locust plantation and the other one was umbrella pine plantation, and identified that although both species had favourable effects on initial soil formation, more rapid accumulation of C and N in the soil profile was registered in black locust forest [15]. Based on Reclaimed Mine Soil Index (RMSI), researchers in India found that tree species growing on reclaimed areas had diverse effects on rhizosphere soil properties [16]. However, little, if any, attention has been paid to explore the coevolution of soil and vegetation to find superior and inferior positions in different reclamation patterns in the Loess Plateau, China. Accordingly, this study was devised to develop scientific and reasonable methods to quantify the interaction between soil and vegetation, and thereby to find the ‘short plank’ (weaknesses) of the sustainable development and guide future ecological reclamation in disturbed areas [17].

Coupling, a common phenomenon stemming from physics, reflects the degree of interaction among two or more systems [18]. Coordination degree mirrors the level of consistency among different systems [19]. Owing to the natural coupling relation between soil and plant in the process of ecological restoration, it is quite suitable to apply a coupling coordination degree model to analyse the interaction between them. In recent years, while it has been applied in ecological recovery, the relevant studies in mine areas are still in shortage. This paper presents a comprehensive index system and uses the coupling coordination degree model to assess the interaction between plant and soil in mined area. The purposes were to: (i) explore the internal advantages and disadvantages in soil–plant system in each reclamation pattern and (ii) quantify the coupling coordination degrees in different reclamation patterns.

## 2. Material and methods

### 2.1. Study area

**Figure 1.**
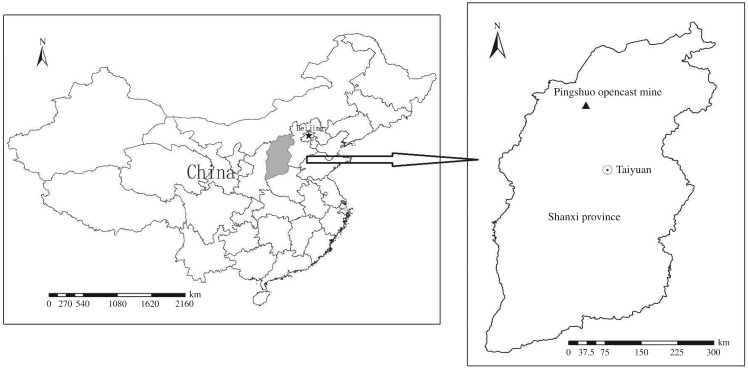
Geographical location of Pingshuo opencast coal mine.

The research was performed in reclaimed coal mine spoils, located in Pingshuo, Shuozhou city, on the border of Shanxi province, Shaanxi province and Inner Mongolia. It is the largest opencast coal mine in China, which has served the mine industry for more than 30 years. The geographical coordinates of the research area range from 112°10′ to 113°30′ E and from 39°23′ to 39°37′ N (figure 1) [20]. The climate is arid to semi-arid continental monsoon climate with a mean annual temperature of 6.4°C and a mean wind speed of 2.5–4.2 m s^−1^ [21]. Mean annual precipitation mainly occurring in summer (from June to September) is 428.2–449.0 mm, while mean annual evaporation is 1786.6–2598.0 mm, almost four to five times larger than the rainfall [22]. According to international soil classification, the dominate soil in the study area is chestnut soil, featured by low organic matter [23].

The original landscape was forest and prairie. However, mining activities had caused destructive effects on the soil physical, chemical and biological properties. From 1993, reclamation measure, to be exact, vegetation reclamation, has been carried out in this region, and by now, an area of 20 km^2^ has been covered with plenty of various plants. The major species in reclaimed forest are *Robinia pseudoacacia*, *Ulmus pumila*, *Ailanthus altissima*, *Pinus tabuliformis*, *Hippophae rhamnoides* and *Caragana korshinskii*.

### 2.2. Sampling plots design

Several 1 ha (100 × 100 m) reclaimed plots with different types of vegetation were permanently established to conduct experiments in summer of 2010, and each plot was then divided into 100 subplots, with an area of 10 × 10 m. Four typical sample plots varied from different plant species were selected in this study. To make a comparison about the coupling coordination degree in reclamation land to that of original land, we surveyed an original landform. All the reclaimed plots were almost similar in elevation, aspect and gradient, and the trees were nearly under the same management. For the first 3 years, plants were watered every year, and the first 5 years, pest control was conducted regularly. The detailed characteristics of each plot are shown in table 1.

**Table 1.** Main features of sampling sites. RUA, *R. pseudoacacia–U. pumila–A. altissima*; RP, *R. pseudoacacia–Pi. tabuliformis*; RM, *R. pseudoacacia* monoculture; PM, *Pi. tabuliformis* monoculture; OP, original *Prunus simonii* monoculture.

plots	years for reclamation	reclamation pattern	vegetation configuration	coordinates	elevation (m)
RUA	23	broadleaved mixed	*R. pseudoacacia–U. pumila–A. altissima*	39°27′42″ N, 112°20′02″ E	1385
RP	23	broadleaved and coniferous mixed	*R. pseudoacacia–Pi. tabuliformis*	39°27′36″ N, 112°19′51″ E	1460.4
RM	23	broadleaved monoculture	*R. pseudoacacia* monoculture	39°27′38″ N, 112°19′39″ E	1436.5
PM	23	coniferous monoculture	*Pi. tabuliformis* monoculture	39°27′40″ N, 112°20′09″ E	1380
OP	―	broadleaved monoculture	*Pr. simonii* monoculture	39°31′42″ N, 112°21′08″ E	1421

### 2.3. Vegetation survey

Vegetation properties in three random subplots were measured to represent each plot in 2016. And each subplot was then subdivided into four 5 × 5 m sub-samples by the interpolation method to get the mean investigated value. In this study, vegetation, including trees, herb and litter, was investigated. For trees, all the free-standing ones with diameter at breast height (DBH) greater than 1 cm were identified and tagged, and their heights (TH) were also recorded. The canopy (TC) of each tree was measured by the line intercept method [24]. Next, TC of each tree was summed, calculated and expressed by percentage to get the total canopy density (TCD) in the whole plot. The biomass (TB) was calculated by allometric equations put forward by Fang *et al.* [25].

In terms of herb, five quadrats (1 × 1 m) were surveyed using a wood frame in each plot to measure coverage (HC), height (HH) and biomass (HB). Litter in each quadrat was collected and weighed to get the biomass (LB).

### 2.4. Soil sampling and analysis

Soil samples from 0 to 20 cm were also collected at the same time. Litter and fermentation in each subplot were first stripped, and five sampling points were selected in each subplot to get a mixed soil sample of 1 kg. A total of 15 samples were collected. For each final sample, after sieving through 2 mm mesh, the soil was kept in No. 8 polyethylene bag and air-dried to test the basic physico-chemical properties.

Soil bulk density (BD) was measured using stainless steel cylinders, and after 48 h oven-dried at 105°C, the ratio of weight and volume of each sample was calculated. pH was measured at a ratio of soil/water of 1 : 1 with an Orion-3 star pH meter [26]. Soil organic matter (SOM) was determined by the Walkley–Black method [27]. Total nitrogen (TN) was measured by the semi-micro Kjeldahl method [28]. Total phosphorus (TP) was analysed by the molybdate colorimetric method after perchloric acid digestion and ascorbic acid reduction [29], and total potassium (TK) by the method of flame atomic absorption spectrophotometer [30]. The alkali diffusion method was used to determine the available nitrogen (AN). Available phosphorus (AP) was measured following the methods of Olsen [31] and available potassium (AK) was determined by flame photometric [32].

### 2.5. Statistical analysis methods

Data preparation and analysis were carried out by Microsoft Excel 2010 and SPSS 16.0 (SPSS, Inc., Chicago, IL, USA). Analytic hierarchy process (AHP) was performed by yaahp v. 6.0.

## 3. Modelling design

### 3.1. The indicator system for a coupling coordination degree model

In this study, we developed soil and vegetation system. For each system, subsystems were further established to gather an in-depth understanding about the mutual effects between soil and vegetation. The indexes in each subsystem were selected according to theoretical analysis and referred to the expert's advice. Final index systems are shown in table 2.

**Table 2.** Index system used to evaluate the relationship between soil and vegetation.

system	subsystem	index
vegetation	tree	canopy density (X1)
		diameter at breast height (X2)
		tree height (X3)
		tree canopy (X4)
		tree biomass (X5)
	herb	herb coverage (X6)
		herb height (X7)
		herb biomass (X8)
	litter	litter biomass (X9)
soil	basic index	pH (X10)
		bulk density (X11)
	nutrient index	soil organic matter (X12)
		total nitrogen (X13)
		total phosphorus (X14)
		total potassium (X15)
		available nitrogen (X16)
		available phosphorus (X17)
		available potassium (X18)

### 3.2. Data pre-processing

The obtained data were expressed by different kinds of units, which made it difficult to process universally. Consequently, data standardization was conducted by the Min–Max standardization method, as shown in the following formula:

3.1 $$X_{ij}^{\prime }=\frac{X_{ij}-\text{min}\{X_{ij}\}}{\text{max}\{X_{ij}\}-\text{min}\{X_{ij}\}}\;,$$

where *X_ij_* represents the obtained value of indicator, and max{*X_ij_*} and min{*X_ij_*} indicate the maximum and minimum value of indicator, respectively.

### 3.3. Determination of index weight

Plenty of subjective and objective methods were set up to ascertain the weight of evaluation index, such as AHP, Delphi, entropy weight method and principal component analysis. Given the influence of the subjective and objective factors, a combined method of AHP and entropy weight method was adopted.

#### 3.3.1. Analytic hierarchy process

AHP is a multi-criterion decision-making method introduced firstly by Saaty [33]. Rather than assigning the weight value directly to the factors, it can evaluate the relative importance of each factor according to the paired comparisons, and a decomposition strategy is used to make it easier and more accurate for decision maker to compare two factors at a time, instead of all the factors simultaneously [34]. In this study, AHP was used to determine the weight of subsystems.

#### 3.3.2. Entropy weight method

Entropy weight method is an objective weight determination method based on the variation of each index, in which information entropy refers to the degree of disorder [35]. For one index, the greater the information entropy, the smaller the variation degree, which means that if the information it can provide is smaller, the weight is accordingly smaller. This method was adopted to determine the weight of indexes in each subsystem. The steps are shown in the following formulae:

3.2 $$P_{ij}=\frac{X_{ij}^{\prime }\;}{\sum_{i=1}^{m}X_{ij}^{\prime }},$$

3.3 $$e_{j}=-\frac{1}{\ln m}\;\overset{m}{\underset{i=1}{\sum }}P_{ij}\;\cdot \;\ln P_{ij}\;(0\leq e_{j}\leq 1)$$

3.4 $$\;\text{and}\;w_{j}=\frac{1-e_{j}}{\sum_{\;j=1}^{n}(1-e_{j})},$$

where *P_ij_* is the proportion of plot *i* in the indicator *j* of all the plots, *e_j_* is the information entropy of indicator *j*, *w_j_* is the weight of indicator *j*, *m* is the number of plots and *n* is the number of indicators in each subsystem.

#### 3.3.3. Combination weight method

An ideal weight evaluation method is to combine the advantages of both subjective and objective methods. Based on the weight of each subsystem and each indicator, a comprehensive weight was calculated by multiplying both of them. The final values are presented in table 3.

**Table 3.** The final weight of each index in the systems.

index	weight	index	weight	index	weight
X1	0.1064	X7	0.0586	X13	0.2272
X2	0.1451	X8	0.0832	X14	0.1141
X3	0.1460	X9	0.1007	X15	0.0427
X4	0.1501	X10	0.0763	X16	0.0688
X5	0.1263	X11	0.1237	X17	0.0833
X6	0.0837	X12	0.1548	X18	0.1092

### 3.4. Comprehensive evaluation function

To reveal the extent of development in each system, we supposed *V_i_* and *S_i_* as composite functions of vegetation and soil system, respectively, with formulae (3.5) and (3.6). The higher the value, the better the development of system.

3.5 $$V_{i}=\overset{a}{\underset{\;j=1}{\sum }}w_{j}\cdot X_{ij}^{\prime }$$

3.6 $$\;\text{and}\;S_{i}=\overset{n}{\underset{j=a+1}{\sum }}w_{j}\cdot X_{ij}^{\prime },$$

where *a* is the number of indicators in vegetation system.

### 3.5. Coupling degree model

Coupling, proposed by Glassman during the research on social livelihood in the 1970s [36], means ‘link connecting two or more parts'. Coupling degree (*C*) mirrors the interaction of two systems, when there exists interworking, there would be benign coupling with a high coupling degree, and vice versa. The computation formula was as follows.

3.7 $$C={\left[\frac{V_{i}\cdot S_{i}}{{\left(V_{i}+S_{i}\right)}^{2}}\right]}^{1/2}.$$

### 3.6. Coupling coordination degree model

While coupling degree shows the interaction intensity among systems, it is not able to reflect the level of coordination, making it an incomplete understanding. But, coupling coordination degree (*D*) can better reflect the degree of harmony between two or more systems [37]. Therefore, *C* was further used to calculate *D*. The process is given in the following formula:

3.8 $$D=\sqrt{C\cdot (\alpha \cdot V_{i}+\beta \cdot S_{i})},$$

where *α* and *β* represent the contribution coefficients of vegetation and soil systems, respectively. In the process of ecological recovery, vegetation and soil were of the same importance; therefore, *α* and *β* were supposed to be equal to 0.5.

For better understanding of the coupling coordination degree, *D* was classified according to previous studies [38,39], the results are given in table 4.

**Table 4.** The classification of coupling coordination degree.

coupling coordination degree (*D*)	coordination degree	*V_i_*/*S_i_*	coordination characteristics
0 < *D* ≤ 0.2	low coordination	0 < *V_i_*/*S_i_* ≤ 0.8	vegetation lagging behind
0.2 < *D* ≤ 0.4	primary coordination		
		0.8 < *V_i_*/*S_i_* ≤ 1.2	synchronous development
0.4 < *D* ≤ 0.6	intermediate coordination		
		1.2 < *V_i_*/*S_i_*	soil lagging behind
0.6 < *D* ≤ 0.8	well coordination		
0.8 < *D* ≤ 1	synchronous development		

## 4. Results and discussion

### 4.1. The evaluation of the ‘short plank’

The recovery of mine area with a fragile eco-environment is an ecological succession of which soil and vegetation are inter-associated and mutually restricted with each other [40]. Considering the principle of ‘short plank’ theory, any lagging behind of the two systems would have a great significance to the whole ecosystem. In this study, comprehensive evaluation function was compared to reveal the development of each system and to further find the ‘short plank’ of each reclamation pattern. According to previous research [19], if the value of *V_i_*/*S_i_* is below 0.8, then the plot is under unbalanced development with soil lagged, otherwise, if the value of *V_i_*/*S_i_* is above 1.2, then the vegetation is the lagging item, and only when the value is just between 0.8 and 1.2, can it be said to have a synchronous development. According to table 5, only plot PM was under synchronous development, RP and RM were vegetation lagged, while RUA and OP were soil lagged. Owing to the restriction of water and other climatic factors, soil in OP was lagged, indicating that the harsh natural environment resulted in an unbalanced development. According to electronic supplementary material, table S2, SOM, AN, AP and AK might be the restraint factors influencing soil development. Thus, artificial measures should be taken to protect and improve the ecological environment to ensure a sustainable use of land. However, mining activities, without doubt, caused serious damage to the already fragile ecosystem [41,42]. Although reclaimed for 23 years, plot of RUA was still developed with soil lagged, manifesting that a mixed model of *R. pseudoacacia*–*U. pumila*–*A. altissima* was not an ideal pattern for soil restoration, and soil properties, especially SOM, TK, AP and AK, in this plot should be given more serious consideration to achieve further coordination development. On the contrary, in RP and RM the development of soil far surpassed that of vegetation. This is mainly associated with biological characteristics of dominant plant species in the reformation of soils [11], because much litter of the *R. pseudoacacia* and *Pi. tabuliformis* can generate a great amount of humus, accelerate nitrogen fixation rate, increase the mineralization rate of carbon and raise other types of nutrients; therefore, the soil quality had been enhanced to a large extent [43,44]. The study by Filcheva *et al*. [45] of reclaimed coal mine spoils planted for 25 years also confirmed the positive impacts of forestation on initial soil-forming processes which encouraged the more rapid transformation of tree litter and created more mobile organic substances that can migrate further into the soil profile. Thus, in these areas, plant growth had been restricted by the natural condition such as shortage of water and illumination. Consequently, reducing the density of plants would be good for advanced development. In plot PM, soil and vegetation were under synchronous development, demonstrating the positive effects of *Pi. tabuliformis* to the modification of soil quality and cooperation between soil and plants; therefore, it can be widely used for the reclamation in this area.

**Table 5.** List of average values of comprehensive evaluation function. RUA, *R. pseudoacacia–U. pumila– A. altissima*; RP, *R. pseudoacacia–Pi. tabuliformis*; RM, *R. pseudoacacia* monoculture; PM, *Pi. tabuliformis* monoculture; OP, original *Pr. simonii* monoculture.

plots	*V_i_*	*S_i_*	*V_i_*/*S_i_*	coordination characteristics
RUA	0.32	0.25	1.26	soil lagging behind
RP	0.57	0.74	0.77	vegetation lagging behind
RM	0.16	0.35	0.45	vegetation lagging behind
PM	0.41	0.46	0.89	synchronous development
OP	0.50	0.21	2.35	soil lagging behind

### 4.2. The evaluation of coupling coordination degree

The purpose of restoration is to achieve the development corresponding to the pre-mining ecosystem; in addition, the coevolution between soil and plants, presented by coupling coordination degree, can determine whether such a reclamation pattern is ecologically feasible or sustainable and further reflect the success of reclamation to some extent [46]. The results of coupling coordination degree in each sampling site are shown in table 6. It can be seen that coupling coordination degrees in five plots varied from 0.34 to 0.57, in the order of RP > PM > OP > RUA > RM and all were under the level of primary and intermediate coordination. Considering that the research area is located in Loess Plateau with an annual precipitation under 500 mm, which mainly occurred in June to September, water should be the biggest limited factor hindering the development of soil and vegetation [22]. Therefore, even plot OP without disturbance was still under primary coordination. With the vegetation reclamation in mined area, properties of soil had been improved, and thus provided more suitable conditions for plant growth, and the coevolution of both soil and plants was largely promoted [47,48]. However, different vegetation patterns have various effects on the improvement of soil environment [49]. In this study, with the succession of plants, the interaction between soil and plants in plots with a mixed model of *R. pseudoacacia*–*Pi. tabuliformis* and *Pi. tabuliformis* monoculture developed prior to those with *R. pseudoacacia*–*U. pumila*–*A. altissima* and *R. pseudoacacia* monoculture, and even more close relationship was found compared with original land, indicating that reclamation patterns of *R. pseudoacacia*–*Pi. tabuliformis* and *Pi. tabuliformis* monoculture were more suitable in this area and the ecological service functions of plants such as nitrogen fixation function and canopy climate regulation in those two plots had been well developed [50]. This was in agreement with Wang *et al.* [51], who did research in species composition in open coal mining area and showed that the vegetation community was stable and the adaptability and survival rate of plants were higher in a mixed model of *R. pseudoacacia*–*Pi. tabuliformis*. This is mainly because *R. pseudoacacia* and *Pi. tabuliformis* can provide a large amount of litter to the soil each year, and then the decomposition can be prompted, resulting in a great improvement in the activities of microbes which couple the soil and plants more intimately [52]. In addition, a mixed model can make good use of the space, lighting, nutrients and so on to exert a complementary effect on different species. While the plot with *R. pseudoacacia* is monocultural, the coupling coordination degree was not wholly satisfactory, mainly due to intraspecific competition of substances necessary for individual growth. And the low diversity of plant leading to an unstable ecosystem could also restrain the development of soil and vegetation. As for plot RUA, the primary coordination is mainly because of the fierce competition among species, and *U. pumila* and *A. altissima* are at an unfair disadvantage, reducing the general coordination degree [53]. Zhang *et al.* [54] also did research in rhizospheric microbial community structure and function during the natural recovery of abandoned cropland and found that increasing competition as more species emerged in the ecosystem caused the complex fluctuations in microbial community composition, which destabilized the decomposition of soil nutrients. All in all, this study may provide basic knowledge for the selection of plant species used for reclamation in refuse dumps in coal mine areas.

**Table 6.** Evaluation results of coupling coordination degree in different plots. RUA, *R. pseudoacacia–U. pumila–A. altissima*; RP, *R. pseudoacacia–Pi. tabuliformis*; RM, *R. pseudoacacia* monoculture; PM, *Pi. tabuliformis* monoculture; OP, original *Pr. simonii* monoculture.

plots	*C*	*D*	coordination degree
RUA	0.4966	0.38	primary coordination
RP	0.4958	0.57	intermediate coordination
RM	0.4632	0.34	primary coordination
PM	0.4992	0.46	intermediate coordination
OP	0.4577	0.40	primary coordination

## 5. Conclusion

Soil rehabilitation and vegetation restoration in mining areas were strongly correlated with each other. Plot of *Pi. tabuliformis* was regarded to have an intermediate coordination degree with synchronous development between soil and vegetation, and a mixed model of *R. pseudoacacia* and *Pi. tabuliformis* was also under intermediate coordination development with soil developed far ahead, while other types of reclamation were not as efficient as those, indicating that the broadleaf conifer mixed forest: *R. pseudoacacia*–*Pi. tabuliformis* and *Pi. tabuliformis* monoculture could be considered as the more suitable patterns for the reclamation in opencast coal mine area of Loess Plateau, China.

## Supplementary Material

Supplementary Material

## Data accessibility

The datasets supporting this article have been uploaded as part of the electronic supplementary material.
