# Supplementary material for: Quantitative correlations between soil and plants in reclaimed mining dumps using a coupling coordination degree model
Source: R Soc Open Sci. 2018 Sep 19;5(9):180484. doi: 10.1098/rsos.180484 (PMC6170530; doi:10.1098/rsos.180484)
Supplement: Supplementary Material [file rsos180484supp1.pdf]

Supplementary Material

Quantitative correlations between soil and plants in reclaimed mining dumps using coupling coordination degree model

Anning Guo,[1] Zhongqiu Zhao,[1,2,*] Ye Yuan,[1] Yangyang Wang,[1] and Xuezhen Li[1], Ruicong Xu[1]

[1]College of Land Science and Technology, China University of Geosciences, Beijing, No. 29, xueyuan Road, Haidian District, Beijing 100083, China.

[2]Key Laboratory of Land Consolidation and Rehabilitation Ministry of Land and Resources, Beijing 100035, China

*Author for correspondence(zhongqiuzhao@163.com)

Present address: College of Land Science and Technology, China University of Geosciences, Beijing, No. 29, xueyuan Road, Haidian District, Beijing 100083, China.

Table S1. Plant properties of all plots

	Trees					Herbs			Litter
Plots	Total canopy density/%	Diameter at breast height /cm	Tree heights/m	Tree canopy/m^2	Tree biomass/g	Herb coverage/%	Herb height/cm	Herb biomass /g	Litter biomass/g
RUA	40.00	6.92	5.25	6.13	39.38	73.33	61.67	210.34	564.16
RP	44.00	11.64	8.46	14.81	58.87	56.67	45.00	97.66	470.34
RM	24.33	8.15	5.30	7.23	33.06	33.33	25.33	99.69	106.40
PM	80.00	7.84	6.80	7.46	66.08	5.00	40.00	7.84	915.96
OP	28.50	13.26	7.47	12.13	80.13	37.50	31.50	54.84	460.95

RUA: *R. pseudoacacia- U. pumila- A. altissima*; RP: *R. pseudoacacia- P. tabuliformis*; RM: *R. pseudoacacia* monoculture; PM: *P. tabuliformis* monoculture; OP: original *P. simonii* monoculture.

Table S2. Soil properties of all plots

Plots	pH	Soil bulk density	Soil organic matter/g·kg^{-1}	Total nitrogen/%	Total phosphorus/%	Total potassium /%	Available nitrogen /g·kg^{-1}	Available phosphorus /g·kg^{-1}	Available potassium /g·kg^{-1}
RUA	8.48	1.42	10.25	0.05	0.04	1.54	39.47	2.67	114.67
RP	8.43	1.47	40.79	0.19	0.06	1.83	31.60	3.77	269.00
RM	8.36	1.46	15.37	0.07	0.05	1.59	37.53	3.20	143.00
PM	8.15	1.53	35.16	0.07	0.05	1.67	33.30	4.50	145.33
OP	8.47	1.37	10.70	0.05	0.05	1.77	12.30	2.00	73.30

RUA: *R. pseudoacacia- U. pumila- A. altissima*; RP: *R. pseudoacacia- P. tabuliformis*; RM: *R. pseudoacacia* monoculture; PM: *P. tabuliformis* monoculture; OP: original *P. simonii* monoculture.
